# Two distinctive energy migration pathways of monolayer molecules on metal nanoparticle surfaces

**DOI:** 10.1038/ncomms10749

**Published:** 2016-02-17

**Authors:** Jiebo Li, Huifeng Qian, Hailong Chen, Zhun Zhao, Kaijun Yuan, Guangxu Chen, Andrea Miranda, Xunmin Guo, Yajing Chen, Nanfeng Zheng, Michael S. Wong, Junrong Zheng

**Affiliations:** 1 College of Chemistry and Molecular Engineering, Beijing National Laboratory for Molecular Sciences, Peking University, Beijing 100871, China; 2 Department of Chemistry, Rice University, 6100 Main Street, Houston, Texas 77005, USA; 3 Department of Chemical and Biomolecular Engineering, Rice University, 6100 Main Street, Houston, Texas 77005, USA; 4 State Key Laboratory of Molecular Reaction Dynamics, Dalian Institute of the Chemical Physics, Chinese Academy of Sciences, Dalian, Liaoning 116023, China; 5 College of Chemistry and Chemical Engineering, Xiamen University, Xiamen, Fujian 361005, China

## Abstract

Energy migrations at metal nanomaterial surfaces are fundamentally important to heterogeneous reactions. Here we report two distinctive energy migration pathways of monolayer adsorbate molecules on differently sized metal nanoparticle surfaces investigated with ultrafast vibrational spectroscopy. On a 5 nm platinum particle, within a few picoseconds the vibrational energy of a carbon monoxide adsorbate rapidly dissipates into the particle through electron/hole pair excitations, generating heat that quickly migrates on surface. In contrast, the lack of vibration-electron coupling on approximately 1 nm particles results in vibrational energy migration among adsorbates that occurs on a twenty times slower timescale. Further investigations reveal that the rapid carbon monoxide energy relaxation is also affected by the adsorption sites and the nature of the metal but to a lesser extent. These findings reflect the dependence of electron/vibration coupling on the metallic nature, size and surface site of nanoparticles and its significance in mediating energy relaxations and migrations on nanoparticle surfaces.

## Abstract (web summary)

Energy migrations at metal nanomaterial surfaces are fundamentally important to heterogeneous reactions. Here, the authors employ ultrafast vibrational spectroscopy to show two distinctive energy migration pathways of monolayer adsorbate molecules on differently sized metal nanoparticle surfaces.

## Graphical abstract

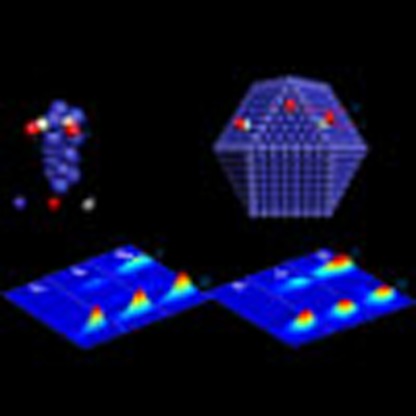

On the surfaces of heterogeneous catalysts, energy dissipation and migration dynamics determine the reaction local temperature and the ability of an adsorbate to cross the reaction barrier. Knowledge about the energy dynamics is essential for a thorough understanding of the selectivity and kinetics of catalytic reactions—and indispensable for developing rational guiding theories of catalyst design^1^,^2^,^3^. However, to experimentally investigate and theoretically describe energy dissipation and migration mechanisms on the surface of a metal nanoparticle, a key component of many important heterogeneous catalysts, remains a grand challenge^1^,^4^,^5^,^6^,^7^,^8^,^9^,^10^. Typically, theoretically modelling a reaction requires modifications of the nuclear potential energy surface with the assumption of separated nuclear and electronic motions during the nuclear movements across its transition state^11^,^12^. This approach, known as the Born–Oppenheimer approximation, which serves as the cornerstone of chemical kinetic theory, allows the electronic energy to be considered independent of the nuclear kinetic energy. Based on the Born–Oppenheimer approximation, the energy (<0.5 eV) released in chemisorption or physisorption of molecules on metal surfaces was usually believed to be dissipated by surface vibrations (phonons), and electronic excitations through electron/vibration coupling were neglected^13^,^14^,^15^,^16^. However, some experimental evidence^17^,^18^,^19^,^20^,^21^,^22^ and theoretical studies^23^ have shown the nonadiabatic effects of vibration/electron coupling on flat metal and some metal nanoparticle surfaces^10^,^24^, questioning the general applicability of the approach. This problem is even more complicated on the surfaces of catalytic metal nanoparticles, as the electronic properties can vary with particle size^25^ and surface sites^26^.

In this work, we demonstrate that the real-time energy migration dynamics between molecules on different surface sites of metal nanoparticles can be monitored with an ultrafast multiple dimensional vibrational spectroscopy. Two distinctive energy pathways are observed for a monolayer of carbon monoxide (CO) molecules adsorbed on different surface sites of a series of platinum (Pt) nanoparticles. On the surface of a 5 nm particle, which is metallic and traditionally theorized to exhibit electron/vibrational coupling^27^, the energy migrations among different sites are very fast, occurring within a few picoseconds. In contrast, the energy migration dynamics between surface sites of small, semi-conductive Pt nanoparticles (∼1 nm clusters) with a much smaller degree of electron/vibrational coupling are more than 1 order of magnitude slower. Further investigations reveal a similar particle-size dependence for the vibrational energy relaxations of the CO molecules: the vibrational relaxations on larger particles (2–11 nm) are also more than 1 order of magnitude faster than those on the 1 nm clusters. Changing the surface adsorption sites and the nature of the metal from Pt to Palladium (Pd) also alters the fast energy relaxation but to a lesser extent compared with the size effect.

## Results

### CO molecules on different surface sites of Pt particles

**Figure 1:**
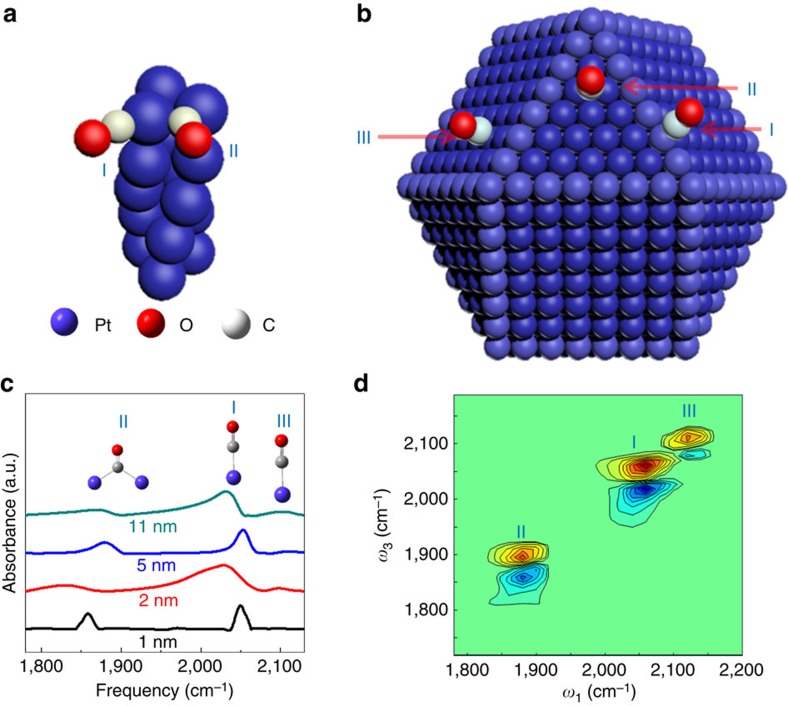
Illustration and spectrums of CO molecules on Pt particles. (**a**) Illustrated CO molecules on the atop (I) and bridge (II) sites of a 1 nm Pt nanoparticle (Pt_15_). In the sample, the actual CO coverage on both surface sites is 100%. (**b**) Illustrated CO molecules on the step (I), bridge (II) and terrace (III) sites of a 5 nm Pt nanoparticle. In the sample, the actual CO coverage on both surface sites is saturated. (**c**) FTIR spectra of CO molecules on Pt nanoparticles in the frequency range of 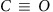 stretch 0–1 transition. (**d**) 2D IR spectrum of CO molecules on 5 nm Pt nanoparticles at zero waiting time, clearly showing the three different CO species (*y*-axis is probe frequency, and *x*-axis is pump frequency). The intensities of peaks II and III and their corresponding blue peaks along the *y*-axis are magnified by 13 and 22 times, respectively, to be comparable to that of peak I.

**Figure 2:**
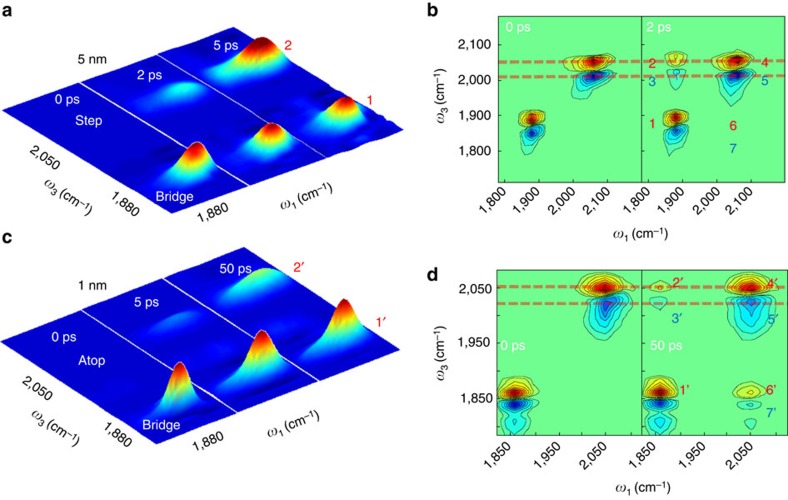
Two distinctive pathways for vibrational energy generation. (**a**) 2D IR spectra of CO stretch (0–1 transition) on 5 nm Pt particles at three waiting times after exciting CO on the bridge site and detecting responses on both bridge and step atop sites. The growth of peak 2 at the step site indicates the generation of heat. (**b**) 2D IR spectra of CO stretch (both 0–1 and 1–2 transitions) on 5 nm Pt particles at two waiting times. The frequencies of the cross peak pairs (peaks 2 and 3) are different from those of the diagonal peak pair (peaks 4 and 5), indicating that peaks 2 and 3 arise because of heat generation. Red dashed lines are drawn to highlight the frequency differences. (**c**) 2D IR spectra of CO stretch (0–1 transition) on 1 nm Pt particles at three waiting times by exciting CO on the bridge site and detecting responses on both bridge and step atop sites. The growth of the peak 2′ at the step site indicates the direct vibrational exchange between CO molecules on the two surface sites. (**d**) 2D IR spectra of CO stretch (both 0–1 and 1–2 transitions) on 1 nm Pt particles at two waiting times. The frequencies of the cross peak pairs (peaks 2′ and 3′) are the same as those of the diagonal peak pair (peaks 4′ and 5′), indicating that peaks 2′ and 3′ are due to direct vibrational transfer between CO molecules on the two sites. The doublet of the blue peak of bridge CO is attributed to a Fermi resonance^57^. (The diagonal red peaks 1 and 1′ are normalized to 1). For the 2D IR spectra, *y*-axis is probe frequency, and *x*-axis is pump frequency.

The samples investigated in this work are 1 (Fig. 1a), 2, 5 (Fig. 1b) and 11 nm Pt clusters and nanoparticles and ∼1 and 5 nm Pd clusters and particles, all of which are coated with a monolayer of CO and ligand molecules (particle characterizations are in Supplementary Figs 1–5). Pt and Pd nanoparticles are important metals in the field of catalysis. They have been used to catalyse the oxidation of CO, aiding in the removal of automobile exhausts, and also serve as classical model systems for mechanistic studies of heterogeneous catalysis for many years^28^,^29^. On the surface of the triangular prismatic 1 nm particle, CO molecules can reside on either the atop site (I) on the top of a Pt atom or the bridge site (II) between two Pt atoms (Fig. 1a). The CO molecules on the atop sites have a CO stretch 0–1 transition frequency at ∼2,047 cm^−1^, and those at the bridge sites have a frequency at ∼1,860 cm^−1^ (Fig. 1c (black curve))^30^. On the surface of 5 nm polyhedral-like Pt nanoparticles (Fig. 1b), CO molecules can occupy the terrace atop site (III), the step atop site (I) and the bridge site (II)^31^,^32^. As displayed in the two-dimensional infrared (2D IR) spectrum in Fig. 1d, CO molecules on site III have a CO stretch 0–1 transition frequency (*ω*_3_, probe frequency) at ∼2,113 cm^−1^. The frequency on site I is ∼2,053 cm^−1^, and that on site II is ∼1,880 cm^−1^(Fig. 1c). The blue peaks underneath the red peaks along the *ω*_3_ axis in Fig. 1d are the CO stretch 1–2 transition peaks. The blue peaks have lower *ω*_3_ frequencies than the corresponding red peaks because of vibrational anharmonicities^33^,^34^,^35^. On the surfaces of other Pt particles (2 and 11 nm), CO molecules can also occupy the atop, bridge and terrace sites. Because the surface structures and the relative surface-site numbers differ on particles of different sizes, the frequency and relative intensity of the CO vibrational peak on each surface site vary with the particle size (Fig. 1c).

### Two distinctive energy migration pathways

We employed two experimental strategies to investigate the energy migration pathways on the surfaces of these Pt nanoparticles by the real-time detection of (a) heat/vibrational energy migration on the nanoparticle surfaces and (b) direct measurements of the CO stretch first excited state population relaxations at different surface sites under various conditions. In doing so, we applied an ultrafast frequency-resolved mid-IR pulse to excite CO molecules on each site to the first vibrational excited state. Another ultrafast mid-IR pulse that covers a frequency range^36^ from ∼1,000 to ∼3,500 cm^−1^ simultaneously detects the optical responses at the exciting frequencies, as well as frequencies associated with molecules on other surface sites. Tuning the waiting time between the excitation and detection pulses, (a) the heat generation was monitored by recording the heat-induced CO stretch 0–1 transition bleaching signals of CO molecules and (b) the direct CO stretch excitation relaxations were monitored in real-time by measuring the decays of the CO stretch 0–1 and 1–2 transition signals. (Signal origins are presented in Supplementary Method).

As displayed in Fig. 2a (where only the 0–1 transition signal is plotted), on the surface of the 5 nm Pt, after the CO molecules on the bridge site II are initially excited by a laser pulse, at zero waiting time, there is only one diagonal peak (peak 1) in the 0 ps spectrum. At 2 ps, a cross peak (peak 2) grows in at 2,062 cm^−1^, ∼9 cm^−1^ higher in frequency than that of the CO 0–1 transition on site I (Fig. 2b). At 5 ps, peak 2 grows in further and surpasses peak 1 in intensity. Depiction of 2D IR approach of the 5 nm Pt sample is shown in Supplementary Fig. 6. In contrast, the growth of the cross peak 2′ on the surface of the 1 nm Pt particles is significantly slower (Fig. 2c,d). At a waiting time of 50 ps, its intensity is still less than 20% of the diagonal peak 1′ (Fig. 2d). The growth of peaks 2 and 3 (Fig. 2b) is caused by the heat generated from the vibrational relaxation of the CO stretch on site II, similar to those in liquid samples described in great detail in our previous work^36^,^37^. The growth of peaks 2′ and 6′ (Fig. 2d) is caused by vibrational energy exchanging between two sites. Illustration of 2D IR approach on the 1 nm Pt sample is shown in Supplementary Fig. 7.

**Figure 3:**
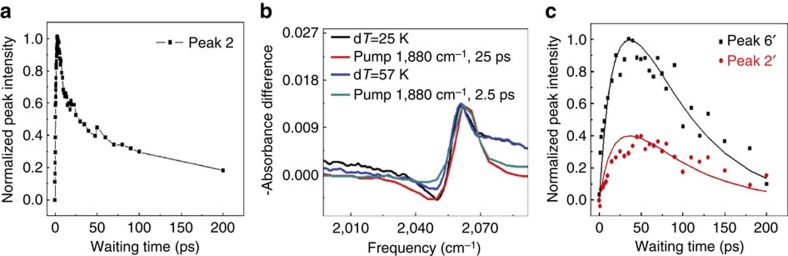
Energy migration induced time-dependent spectrum dynamics. (**a**) The time-dependent intensities of peak 2 (*ω*_1_=1,880 cm^−1^, *ω*_3_=2,062 cm^−1^) on the 5 nm Pt nanoparticle. (**b**) Comparison of temperature difference FTIR (black and blue curves) and 2D IR data at waiting times 2.5 ps (red) and 25 ps (cyan). Intensities were normalized to similar values for better illustration. (**c**) The time-dependent intensities of the cross peak signals 6′ (*ω*_1_=2,047 cm^−1^, *ω*_3_=1,860 cm^−1^) and 2′ (*ω*_1_=1,860 cm^−1^, *ω*_3_=2,047 cm^−1^) on the 1 nm Pt nanoparticle.

The particle size-dependent responses of the step site CO indicate different energy migration pathways. Recent work^25^,^38^ suggests that the electronic properties of metal nanoparticles are dictated by the number of atoms and thus the particle size. For example, 5 nm polyhedral particle is metallic^39^,^40^ and exhibits a continuum of electronic states. As a result, theoretical studies predict that the conduction electrons on these metallic Pt nanoparticle surfaces can be excited from just below to just above the Fermi level (excitation of an electron/hole pair) by the resonant energy transfer from the CO stretch vibrational excitation^27^. The electron/hole pairs excited by the vibrational relaxation can quickly recombine and release their energy to the lattice motions (phonons, or ‘heat') within 1∼2 ps, and quickly migrate on the surface, raising the metal surface temperature^41^,^42^. As a result of the temperature increase, the CO stretch 0–1 transition frequency (2,053 cm^−1^) on site I red-shifts, which is corroborated by the temperature-dependent Fourier transform infrared spectroscopy (FTIR) spectra (in Supplementary Fig. 8). Such a red shift produces a bleaching signal at a frequency (2,062 cm^−1^) higher than 2,053 cm^−1^ and a new absorption at a frequency (2,047 cm^−1^) lower than 2,053 cm^−1^ in the 2D IR spectra (Fig. 2b). The excitation frequency (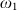
=1,880 cm^−1^) of peaks 2 and 3 reflects the origin (site II) of the energy dissipation process, and the respective detection frequencies (*ω*_3_=2,062 and 2,047 cm^−1^) demonstrate the spectral changes of the CO stretch on site I caused by the process. Therefore, how the intensities of the two peaks change with the waiting time is indicative of thermal energy migration dynamics from site II to site I. As displayed in Fig. 3a, peak 2 grows very fast and reaches a maximum at 2∼3 ps and then decays slowly, suggesting that the thermal energy migrates from site II to site I very rapidly within 2∼3 ps and then dissipate to environment slowly. By comparing the 2D IR results to the temperature difference FTIR spectra (Fig. 3b), the temperature increase on site II within the initial 2∼3 ps is found to be >57 K (Fig. 3b), and the increase drops to about 25 K at a delay of 25 ps after the thermal energy has further dissipated to the environment. The spectral changes caused by the temperature increase are very small in the frequency range of bridge site CO stretch (∼1,880 cm^−1^) (Supplementary Fig. 8b). The additional overlap, occurring with the tail of a very strong 1–2 absorption signal of the step atop site CO stretch after 2 ps (Fig. 2b), makes the heat-induced cross peaks around 6 and 7 (*ω*_1_=2,053 cm^−1^, *ω*_3_=1,880 cm^−1^) too weak to be observed.

In contrast to peaks 2 and 3 of the 5 nm Pt particle, the growth of peaks 2′ and 3′ are not caused by the migration of thermal energy generated from vibrational relaxation, but by the direct vibrational energy exchange between sites I and II CO as their frequencies are identical to those of the diagonal peaks 4′ and 5′ (Fig. 2d). The growth of the cross peaks is not caused by chemical exchange, in which case the peak growth rate constant ratio of 6′ over 2′ would be equal to 1 (ref. 35). The 1 nm cluster (H_2_[Pt_3_(CO)_3_(μ_2_-CO)_3_]_5_) is a semiconductor with a HOMO-LUMO energy gap of ∼1.7 eV (see visible spectrum in Supplementary Fig. 1d)^43^,^44^. This energy splitting is considerably larger than the CO vibrational 0–1 transition energy of 0.25 eV, and thus the CO stretch vibrational energy cannot excite the Pt electrons from the HOMO to the LUMO. Therefore, the CO vibrational energy on 1 nm sample cannot be mediated by electronic excitation, it can only transfer directly to the bridge site (II) C–O stretch (peaks 2′ and 6′) via vibration/vibration coupling. When direct vibrational energy exchange occurs, the population ratio is determined by detailed balance (this system is 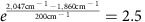)^45^. Kinetic analyses show that the peak growth rate ratio (Fig. 3c) is also 2.5 (detailed kinetic analyses are found in Supplementary Note 1). No heat signal is observed up to 50 ps (Fig. 2d) (The control temperature-dependent FITR of 1 nm sample is shown in Supplementary Fig. 9). This result indicates that the heat generation induced by CO vibrational relaxation on the 1 nm Pt particle is significantly slower than 50 ps, which is already more than 20 times slower than on the 5 nm Pt particle.

### Two distinctive energy dissipation pathways

**Figure 4:**
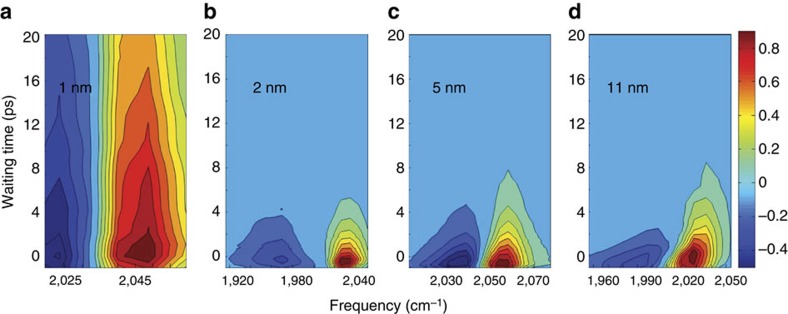
Time-dependent spectrum of CO on Pt nanoparticles. The waiting time-dependent CO stretch 0–1 (red) and 1–2 (blue) transition signals of CO molecules on the atop step site of (**a**) 1 nm Pt particles, (**b**) 2 nm Pt particles, (**c**) 5 nm Pt nanoparticles and (**d**) 11 nm Pt nanoparticles after these molecules are excited to the CO stretch first excited state. The signal from the 1 nm particle lasts significantly longer than the signal from larger particles.

The distinct energy migration pathways following the surface molecular vibrational excitations on the two sizes particles are further supported by direct measurements of the CO stretch first excited state population relaxations at different surface sites under various conditions. Figure 4 displays the waiting time-dependent CO stretch 0–1 (red) and 1–2 (blue) transition signals of CO molecules on the step atop site of 1, 2, 5 and 11 nm Pt particles after excitation to the first excited state of the CO stretch. On the surface of 1 nm Pt particles (Fig. 4a), after 20 ps, both 0–1 and 1–2 transition signals still remain >50% of the initial intensity. However, on larger particles, the signals decay much faster. Within 4 ps more than 80% of the signal has decayed (Fig. 4b–d). This demonstrates that the CO vibrational energy dissipates much faster on the surfaces of larger nanoparticles. This fast relaxation is consistent with the observed rapid heat generation from CO relaxation on step sites of metallic nanoparticles. From Fig. 4b–d, we can also see that the 0–1 transition signals slightly blue-shift with the increase of waiting time and last a little longer than the 1–2 transition signals, which is similar to Fig. 2c.

**Figure 5:**
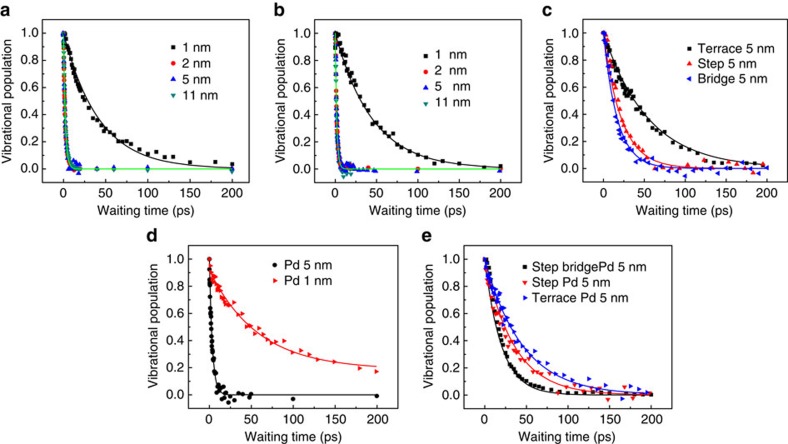
Vibrational relaxation of CO in different conditions. (**a**) Vibrational relaxations of CO at the step site (I) on differently sized Pt particles; (**b**) Vibrational relaxations of CO at the bridge site (II) on differently sized Pt particles; (**c**) Vibrational relaxations of CO on the 5 nm Pt particle at different sites; (**d**) Vibrational relaxations of CO on the step atop site of 5 nm Pd particles and the atop site of 1 nm Pd (Pd/Ag) particles; (**e**) Vibrational relaxations of CO on 5 nm Pd particles at different sites. Experimental data points are fit with curves.

Quantitative analyses (Fig. 5a) of the data show that the CO stretch first excited state vibrational energy decays with a time constant of 42±5 ps on site I of the 1 nm particle. In contrast, the relaxations time constants are more than 15 times faster and identical on the same site of the 3 larger-sized Pt particles, 2.2±0.2 ps. Similarly, particle size dependence of the CO vibrational relaxations is observed on the bridge site (II) (Fig. 5b). The CO stretch first vibrational excited state population decays with a time constant of 47±5 ps on the bridge site of 1 nm particles. On the same surface site of the larger (2–11 nm) Pt particles, the decay is significantly faster with a time constant of 1.8±0.3 ps, which is consistent with fast temperature rise in Fig. 3a. In addition, the fast relaxation dynamics on step site CO are independent (within the experimental uncertainty ∼10%) of isotopic labelling ^13^C^18^O (Supplementary Fig. 10a ), temperature variance (293–80 K) (Supplementary Fig. 10b) and environmental modifications (Supplementary Fig. 10c). In contrast, without the surface electronic excitation, the CO relaxation on 1 nm Pt particle surfaces follows a similar mechanism (vibration/vibration coupling) as the metal carbonyl small molecules^46^, 1–2 orders slower than the dynamics observed on metallic nanoparticle surfaces.

The fast CO stretch vibrational decays on metallic Pt particles surfaces (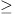
2 nm) were also observed on other surface sites besides sites I and II. Close examination of the rates reveals that different kinds of binding at the surface sites result in different dynamics. As shown in Fig. 5c, the CO vibrational energy relaxation on the terrace site (III) has a time constant 5.5±0.6 ps. The relaxations (1.8±0.3, 2.2±0.2 and 5.5±0.6 ps) on all three surface sites are of the same order time scale and all significantly faster than the dynamics observed on the 1 nm Pt particle surface (40–50 ps). The observed metal/nonmetal dependence of CO relaxations is similar to that of previous experiments^10^,^24^. However, probably due to technical limitations, previous experiments did not observe the surface-site dependence of the CO lifetimes and the CO lifetime on the step atop site determined then was too long (∼7 ps)^10^,^24^. The CO relaxation on larger particle sizes in this work showed clear site dependence. The energy dissipation is slightly faster on the bridge site than the terrace atop site, and it is faster on the step atop site than the terrace atop site. According to previous theoretical study^27^, vibrational relaxations induced by electron/vibration coupling are determined not only by the available electronic energy levels but also by the coupling strength between the surface electrons and the chemical bond that vibrates. The available energy levels of surface electrons are identical for each surface site. And, thus, the experimental results suggest that the CO/surface electron coupling strengths vary on different surface sites of nanoparticle, consistent with the different binding strength of CO molecules on distinct surface sites from previous studies on Pt flat surface^26^,^47^.

### Particle-size and surface-site dependences on Pd surfaces

Similar trends are also observed on the surfaces of Pd nanoparticles. As displayed in Fig. 5d, the CO stretch vibrational relaxation (3.5±0.5 ps ) on the step atop site of 5 nm Pd metallic particles is about 20 times faster than that (67±15 ps) on the atop site of ∼1 nm Pd semi-conductive clusters (Pd/Ag bimetallic particle <2 nm). The relaxation times follow the same relative order as those of metallic Pd particles: bridge (2.0±0.2 ps)<step atop (3.5±0.5 ps)<terrace atop (6.1±0.5 ps) (<<1 nm (67±15 ps)) (Fig. 5e). Comparison of the 5 nm Pd to the 5 nm Pt shows the CO stretch vibrational relaxation on Pd is slightly slower than that on Pt at each surface site. This is probably because CO/Pt interactions are slightly stronger than CO/Pd interactions^48^.

## Discussion

Two distinct energy migration pathways on nanoparticle surfaces were observed. On a large metallic nanoparticle, the vibrational energy of a surface molecule can rapidly dissipate into the particle through electron/hole pair excitations. The electron/hole pairs can then quickly recombine and release their energy to the lattice motions (phonons, or ‘heat'), and quickly migrate on the surface, raising the surface temperature. All the processes occur within a few picoseconds. On a small and semiconducting particle, because of the lack of electron/vibration coupling, the surface vibrational relaxation and the energy migration between surface molecules are mainly through the vibration/vibration coupling mechanism and are >1 order of magnitude slower. Systematic studies also reveal that the electron/vibration coupling is little dependent on temperature (from 80 to 293 K) or the particle size only if the particle is metallic, but is dependent on the particle surface site and the nature of the metal. The findings may stimulate further application and development of existing nonadiabatic theory to explore the dependences^23^.

## Methods

### Materials and synthesis procedures

The chemicals used for synthesizing the nanoparticles were purchased from Sigma-Aldrich and used without further purification and the CO gas was purchased from Matheson and Sigma.

### Preparation and characterization of 1 nm Pt nanoparticle

About 0.5 ml (0.1 M) aqueous solution of chloroplatinic acid (H_2_PtCl_6_·6H_2_O) dissolved in 10 ml dimethylformamide (DMF) can be sealed into 1.5 atm CO glass vessel for 28 h to obtain the blue–green sample. The structure of the cluster was characterized with mass spectrometry^43^. In the DMF solution (Fig. 1b and Supplementary Fig. 1a), the atop site CO absorption is at 2,047 cm^−1^, and the bridge site CO absorption is around 1,860 cm^−1^. The FTIR spectrum in Fig. 1c multiplies the intensities of the atop CO and bridge CO absorption regions by 25 times for better illustration. The IR spectrum (Supplementary Fig. 1c) showed in solid phase also confirmed two main peaks of this sample. The 1 nm Pt particles are unstable in the solid state. All ultrafast measurements presented in this paper were conducted in the DMF solution and finished within 2–3 h. The ultraviolet–visible spectra of the reaction solution before and after the reaction were recorded using a Varian Cary 5000 UV-visible spectrometer. Compared with the H_2_PtCl_6_ solution, two new peaks (Supplementary Fig. 1d) at 400 and 630 nm appear in the cluster solution, consistent with those of the [Pt_3_(CO)_3_(μ_2_-CO)_3_]_5_^2−^. The 1.7 eV energy gap is calculated according to the ultraviolet–visible spectrum because this sample's electronic properties are comparable to a quantum dot.

### Preparation and characterization of 2 nm Pt nanoparticle solid sample

K_2_PtCl_4_ (3 mM ethylene glycol solution) and 10 mM sodium acetate were mixed in hot 1, 2-ethanediol at 80 °C, stirred for 30 min. Thus, ∼2 nm Pt nanoparticles were synthesized following the reported method^49^. The hot solution was then cooled down. CO gas was bubbled at the rate of 200 ml min^−1^ within 30 min in a hood. The solution was then mixed with water at a v/v ratio 3:1. The mixture was centrifuged for three times with 26,000 r.p.m. for 38 h or 40,000 r.p.m. for 20 h. The precipitate was suspended in several drops of 1,2-ethanediol. The mixture was dropped onto CaF_2_ windows and transferred into the vacuum oven overnight to remove solvent. The sample-covered CaF_2_ window was placed into vacuum chamber and oil-free pumped overnight. We applied the same procedures to bubble Pt nanoparticles (2, 5 and 11 nm) and Pd nanoparticles (1 and 5 nm) samples, prepared the solid samples in vacuum chamber, measure the solid samples FTIR spectrum and collect ultrafast data. The frequency ranges (1,830–1,890 cm^−1^) and (2,090–2,130 cm^−1^) of FTIR Fig. 1c of 2 nm sample in the result part were zoomed in three times for better illustration. The sample was characterized by JEM 2100F TEM and Rigaku D/Max Ultima XRD. The transmission electron microscopy (TEM) and XRD data are shown in Supplementary Fig. 2a,b (high resolution) and Supplementary Fig. 3a. The temperature control measurements were performed with JANIS model ST-100.

### Preparation and characterization of 2 nm Pt nanoparticle solution sample

K_2_PtCl_4_ (0.0174, g, 4.2 × 10^−5^ mol), poly(acrylic acid sodium salt) (molecular weight (MW)∼5,100, 0.0394, g) and 10 ml ethylene glycol were mixed in a 50 ml flask. It is noted that poly(acrylic acid sodium salt) shows poor solubility in ethylene glycol at room temperature. Under vigorously stirring, the solution was heated to 180 °C and kept at 180 °C for 10 min. The colour of solution would become dark and clear with increasing temperature, which indicates the formation of ∼2–3 nm Pt NPs. Then, the sample was dropped on the CaF_2_ window. Vacuum pump was used to remove main parts of ethylene glycol solvents in the vacuum oven. Only two drops of high-concentration solution were left on the CaF_2_ window. Another CaF_2_ was used to quickly seal the sample between two CaF_2_. This solution sample did not need to be transferred into vacuum chamber for measurement. The FTIR is shown in Supplementary Fig. 3b. The method to prepare isotope-labelled 2 nm Pt was exactly same as that for the powder 2 nm Pt NP sample. K_2_PtCl_4_ (3 mM ethylene glycol solution) and 10 mM sodium acetate were mixed in hot 1, 2-ethanediol at 80 °C, stirred for 30 min. The isotope ^13^C^18^O gas 0.5 l (purchased from Sigma) was bubbled into the solution. Samples were then prepared according to the procedure described above.

FTIR spectra of CO and ^13^C^18^O on the 2 nm Pt nanoparticles were shown in Supplementary Fig. 3c. The vibrational decay data of the ^13^C^18^O stretch excitation in this paper were obtained by exciting the shoulder at ∼1,940 cm^−1^. The 2D IR of this sample is shown in Supplementary Fig. 3d. The blue peak around (1940cm^−1^, 1870, cm^−1^) stems from the ^13^C^18^O 1–2 transition and indicates its binding formation on the step site.

### Preparation and characterization of 5 nm Pt nanoparticle

Pt(II) acetylacetonate (80 mg, ∼0.2 mmol) and polyvinylpyrrolidone (PVP, 55 mg, MW=55,000) were dissolved in 5 ml of ethylene glycol. The solution was then heated to 200 °C in a microwave reactor (Anton Paar Monowave 300) under a stirring rate of 1200, r.p.m. min^−1^ and held at that temperature for 5 min and then cooled to room temperature. The as-prepared Pt nanoparticles were precipitated with 45 ml of acetone and re-dispersed in 10 ml of ethylene glycol to remove excess PVP. Thus, the 5 nm Pt nanoparticles were synthesized according to the reported method^50^. The TEM image is shown in Supplementary Fig. 2c. The original FTIR intensities of the bridge and terrace are weak. In the Results part, FTIR Fig. 1c bridge area (1,830–1,890 cm^−1^) and terrace area (2,090–2,130 cm^−1^) of 5 nm sample were zoomed in 10 times for better illustration.

### Preparation and characterization of 11 nm Pt nanoparticle

First, 250 ml 1 × 10^−4^ M K_2_PtCl_4_ solution in DI water was prepared, followed by adding 0.2 ml of 0.1 M sodium polyacrylate. Then N_2_ gas (>99.99%, Matheson) was bubbled into the mixture at a flow rate of 200 ml min^−1^ for 20 min. Reduction of Pt ions was started by switching the bubbling gas to H_2_ (>99.99%, Matheson) for 10 min at a flow rate of 200 ml min^−1^. The reaction vessel was then sealed and left under room temperature for 48 h to complete the reduction. Thus, the preparation method of the 11 nm Pt nanoparticles in this study followed the method by Ahmadi *et al*.^51^ The TEM picture is shown in Supplementary Fig. 2d.

### Preparation and characterization of 5 nm Pd nanoparticle

About 0.449 (2.00 mmol) of palladium(II) acetate and 0.85 g of PVP (MW=55,000) were dissolved in 20 ml of 2-ethoxyethanol. The solution was then heated to 110 °C in a microwave reactor (Anton Paar Monowave 300) under a stirring rate of 1,200 r.p.m. min^−1^ and held at that temperature for 60 min, and then cooled to room temperature. The as-prepared Pd NPs are used for spectroscopic measurement. The TEM picture is shown in Supplementary Fig. 2e. The FTIR of this sample is shown in Supplementary Fig. 4.

### Preparation and characterization of 1 nm Pd–Ag nanoparticle

The preparation method was a revised method for the preparation of Ag_44_ (ref. 52). Supplementary Fig. 5a briefly presented the synthesis procedure. In detail, 0.25 mmol of silver nitrate (AgNO_3_) and 0.0625, mmol of palladium acetate (Pd(O_2_CCH3)_2_) were dissolved in 21 ml water and 1.25 mmol of para-mercaptobenzoic acid (p-MPA) were dissolved in 12 ml ethanol. The aqueous solution and ethanolic solution were mixed to form a yellowish insoluble precursor. The pH was then raised to 12 to deprotonate p-MPA by using aqueous CsOH (50% w/v). The colour of solution immediately became red with the increase of pH value. After that 9 ml of aqueous NaBH_4_ (3.125 mmol) was slowly added to the precursor solution with vigorously stirring. The colour of solution turned to dark after adding NaBH_4_ solution. After 1 h reaction, the obtained product was cleaned first by centrifuging to remove any solid and then precipitating the clusters with DMF to remove salts and other left-over soluble materials from the reaction. TEM picture is shown in Supplementary Fig. 5b and X-ray photoelectron spectroscopy (XPS) is shown in Supplementary Fig. 5c. The ratio of Pd/Ag/S equals 1:6.2:6.8, calculated from the peak area/relatively sensitivity factor with the software Multipak of the XPS machine. Thus, the as-prepared nanoparticle is the Pd–Ag bimetallic structure. The low metal/ligand ratio suggested the small size of this particle. The multi-absorbance peaks in ultraviolet–visible spectrum also indicate that the size of Pd–Ag bimetallic nanoparticles is <2 nm—otherwise, a strong surface Plasmon resonance peak from Ag nanoparticles would be observed at ∼420 nm (ref. 53). The TEM image (Supplementary fig. 5b) also suggests that the size is very small (<2 nm) and results in poor image resolution. Because the binding strength of CO with Pd is much stronger than that of CO with Ag (refs 54, 55), the CO molecules on the Ag–Pd bimetallic nanoparticle dominantly reside on Pd atoms.

### Optical methods

Briefly, the same seed pulse is employed to synchronize a picosecond amplifier and a femtosecond amplifier. The two amplifiers are then used to produce pump and probe IR beams, respectively. The ∼0.8 ps mid-IR pulses (vary from 0.7 to 0.9 ps according to the frequency) were generated by the ps amplifier pumping an optical parametric amplifier. The bandwidth of the 1 kHz mid-IR beam as pumping beam in measurement was around 10–35 cm^−1^ in a tunable frequency range of 400 to 4,000 cm^−1^ with energy ranging from 1∼ 40 μJ per pulse (>10 μJ per pulse for 1,000–4,000 cm^−1^). The probing beam (a high-intensity mid-IR and terahertz super-continuum pulse with a duration of <100 fs between 400 to 3,000 cm^−1^ at 1 KHz) was generated from the fematosecond amplifier. Two polarizers are inserted into the probe beam pathway before and after the sample, respectively, to selectively measure the parallel or perpendicular polarized signal relative to the excitation beam. In IR pump/IR probe experiments, the picosecond IR pulse is the pump beam and the super-continuum pulse is the probe beam. Two polarizers are inserted into the probe beam pathway to selectively measure the parallel or perpendicular polarized signal relative to the excitation beam. To avoid the scattering signal (especially for the weak signals at the bridge site and terrace atop site), we used data from the perpendicular configuration rather than the usual rotation-free data to describe the vibrational decay^56^. This approach can introduce some uncertainty in the vibrational lifetimes as resonant vibrational energy transfers and rotation can occur. To address this issue, for both 1 and 5 nm Pt samples, we measured both rotation free (parallel signal plus two perpendicular signals) and perpendicular signals. The data are plotted in Supplementary Fig. 11 and show that the decays from the perpendicular measurements are about 10% slower than the rotation-free data. Because our experimental data have ±10% uncertainty, the data of the isotope labelling ^13^C^18^O, the data do not present a clear difference with the un-labelled data. In all our 2D IR spectra in this paper, the probe frequency resolution is around ±4 cm^−1^ and the pump frequency resolution is ∼20 cm^−1^. The FTIR spectral resolution is around 2 cm^−1^. The control experiment of heat generation of peak 2 in Figs 2 and 3 is introduced in Supplementary Fig. 12. Our signal origins of both diagonal peaks and cross peaks are presented in Supplementary Method.

## Additional information

**How to cite this article:** Li, J. *et al*. Two distinctive energy migration pathways of monolayer molecules on metal nanoparticle surfaces. *Nat. Commun.* 7:10749 doi: 10.1038/ncomms10749 (2016).

## Supplementary Material

Supplementary Information Supplementary Figures 1-12, Supplementary Note 1, Supplementary Methods and Supplementary References
