# Supplementary material for: Two distinctive energy migration pathways of monolayer molecules on metal nanoparticle surfaces
Source: Nat Commun. 2016 Feb 17;7:10749. doi: 10.1038/ncomms10749 (PMC4757789; doi:10.1038/ncomms10749)
Supplement: Supplementary Information — Supplementary Figures 1-12, Supplementary Note 1, Supplementary Methods and Supplementary References [file ncomms10749-s1.pdf]

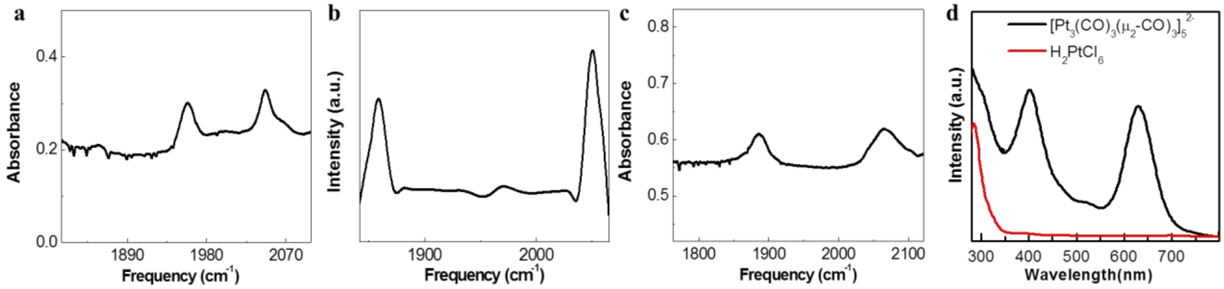

Supplementary Figure 1: Spectra Characterization of 1nm Pt sample. (a) FTIR spectrum of 1nm Pt nanoparticle in DMF solution (40-50um spacer); (b) FTIR spectrum of 1nm Pt nanoparticle in DMF solution without the DMF background (regions between 1846cm^{-1}-1870cm^{-1} and 2042cm^{-1}-2061cm^{-1} are multiplied by 25 for better illustration); (c) FTIR spectrum of the 1nm Pt nanoparticle in solid phase; (d) UV-visible spectrum of 1nm Pt nanoparticle in DMF solution.

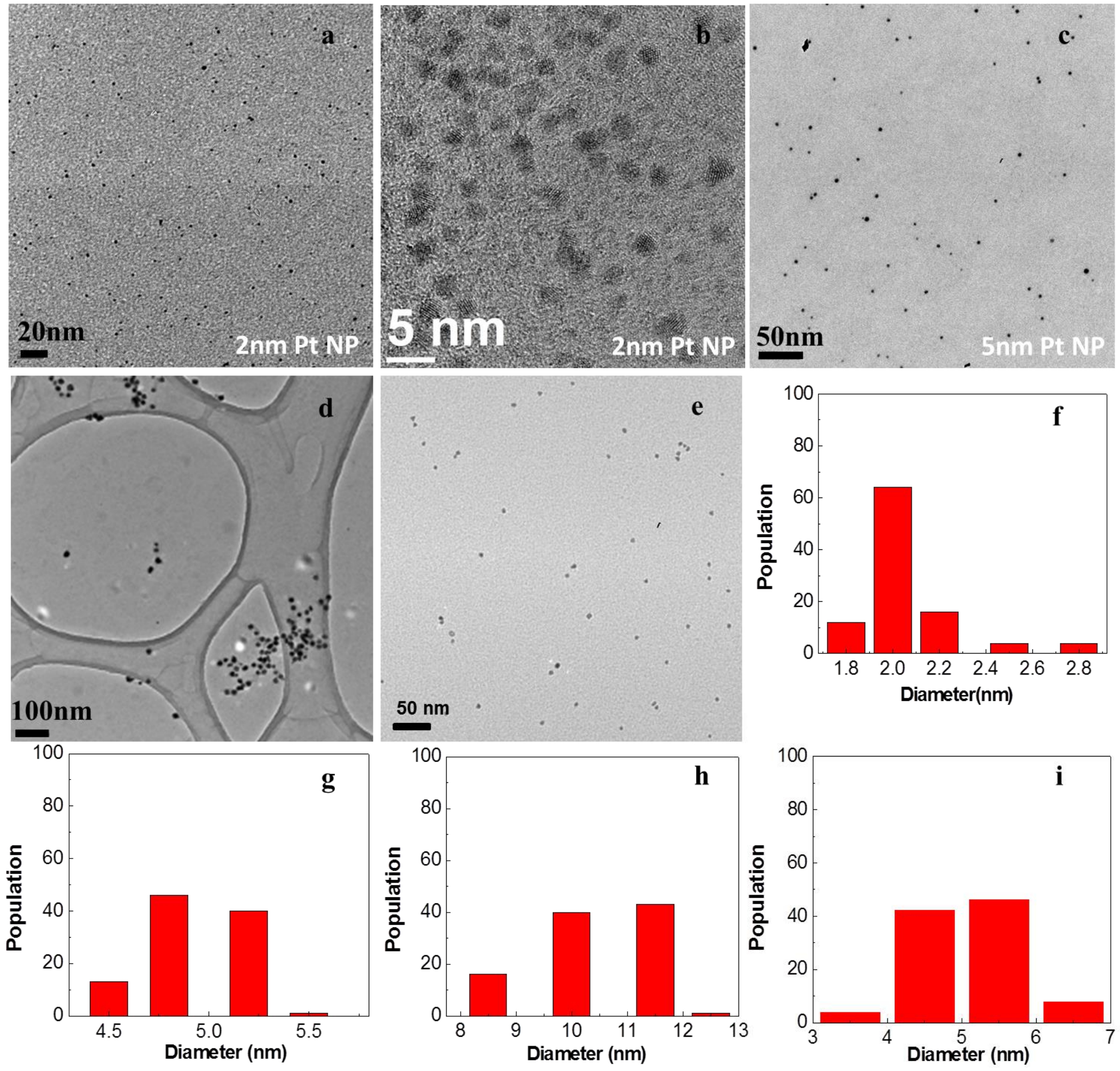

Supplementary Figure 2: TEM Characterization of Pt and Pd nanoparticles. (a) TEM picture of ~2nm Pt nanoparticle; (b) High resolution TEM picture of ~2nm Pt nanoparticle; (c) TEM picture of ~5nm Pt nanoparticle; (d) TEM picture of ~11nm Pt nanoparticle; (e) TEM picture of ~5nm Pd nanoparticle;(f) Histograms of ~2nm Pt nanoparticle based on figure (a); (g) Histograms of ~5nm Pt nanoparticle based on figure (c); (h) Histograms of ~11nm Pt nanoparticle based on figure (d); (i) Histograms of ~5nm Pd nanoparticle based on figure (e).

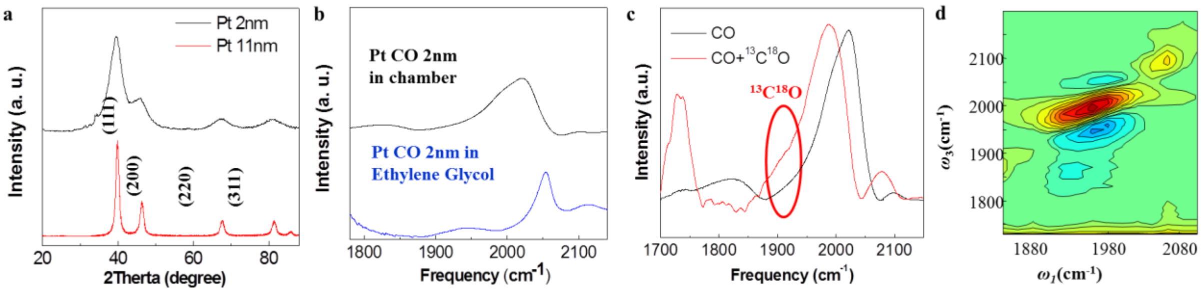

Supplementary Figure 3: Characterization of Pt samples in different conditions. (a) XRD spectra of Pt nanoparticles; (b) FTIR of CO on 2nm samples in solid powder (black) and in solution (blue); (c) FTIR spectrum of CO binding on 2nm Pt sample (Black) and $^{13}C^{18}O$ plus CO binding on 2nm Pt sample (red);(d) 2DIR of CO and $^{13}C^{18}O$ binding on 2nm Pt sample.

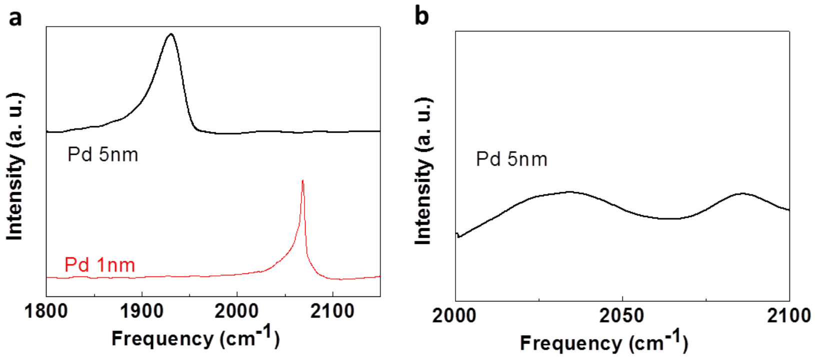

Supplementary Figure 4: Spectra Characterization of the Pd samples. (a) FTIR spectrum of 1nm and 5nm Pd powder samples; (b) FTIR spectrum of 5nm Pd powder sample in the frequency range 2000 ~ 2100 cm^{-1}.

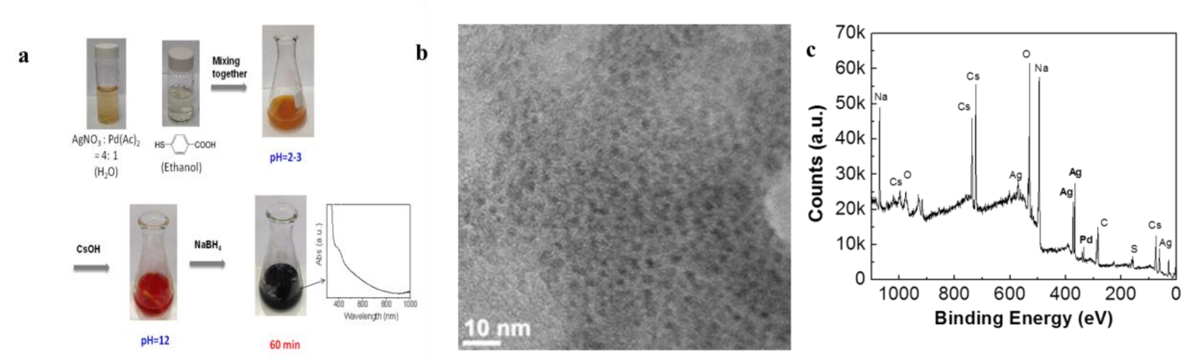

Supplementary Figure 5: Characterization of 1nm Pd sample. (a) Synthesis of ~1 nm PdAg nanoparticle; (b) TEM of the Pd-Ag sample; (c) XPS data of the ~1nm Pd-Ag sample.

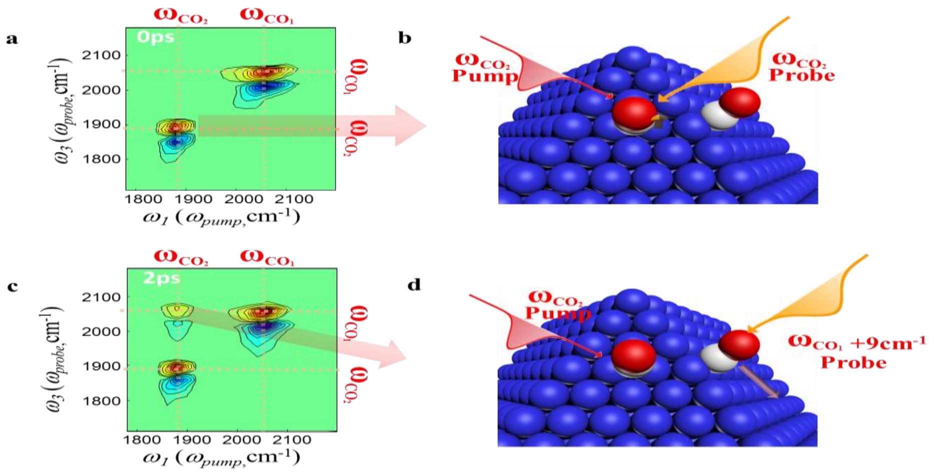

Supplementary Figure 6: Depiction of 2DIR approach as applied to the 5nm Pt sample. (a) 2DIR spectrum collected at a time delay of 0 ps. $\boldsymbol{\omega}_{CO_1}$ and $\boldsymbol{\omega}_{CO_2}$ are 0-1 transiton frequencies of CO molecule strech modes on the step atop and bridge sites, respectively. The x-axis is the pumping frequency, and the y-axis is the probing frequency. (b) Representation of the peak ($\boldsymbol{\omega}_{CO_2}$,$\boldsymbol{\omega}_{CO_2}$) in supplementary figure 6a. The pumping IR light with frequency $\boldsymbol{\omega}_{CO_2}$ selectively excites the CO strech mode on the bridge site from the ground state to the 1st excited state, and the broadband probing beam with $\boldsymbol{\omega}_{CO_2}$ detects the population of CO on the1st excited state. (c) 2DIR spectrum collected at a time delay of 2 ps. (d) Representation of the peak ($\boldsymbol{\omega}_{CO_2}$,$\boldsymbol{\omega}_{CO_1}$+9cm^{-1}) in supplementary figure 6c.The pumping IR light with frequency $\boldsymbol{\omega}_{CO_2}$ selectively excites the CO strech mode on the bridge site from the ground state to the 1st excited state, because of fast energy relaxation, local temperature of CO on step sites increases, and creates some excitation of low frequency modes (for example, CO translational motion on the Pt surface and surface Pt-Pt lattice motion). This produces a red shift of the CO step site spectrum and the induced bleaching signal at $\boldsymbol{\omega}_{CO_1}$ +9cm^{-1}. The broadband probing beam at a frequency of $\boldsymbol{\omega}_{CO_1}$ +9cm^{-1}detects the induced bleaching signal.

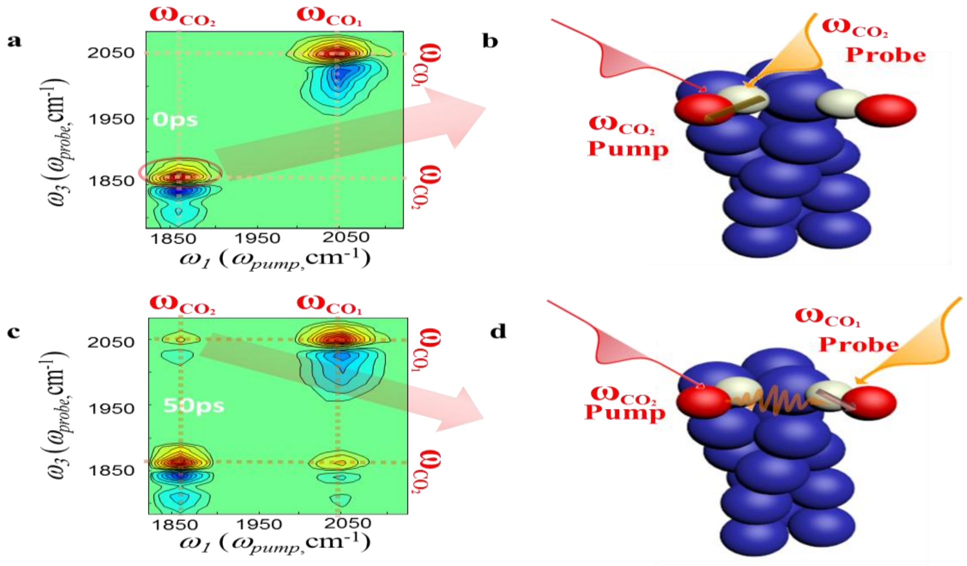

Supplementary Figure 7: Depiction of 2DIR approach as applied to the 1nm Pt sample.(a) 2DIR spectrum collected at a time delay of 0 ps. $\boldsymbol{\omega}_{CO_1}$ and $\boldsymbol{\omega}_{CO_2}$ are 0-1 transiton frequencies of CO molecule strech modes on the step atop and bridge sites, respectively. The x-axis is the pumping frequency and the y-axis is the probing frequency. (b) Representation of the peak ($\boldsymbol{\omega}_{CO_2}$,$\boldsymbol{\omega}_{CO_2}$) in supplymental figure 7a. The pumping IR light with frequency $\boldsymbol{\omega}_{CO_2}$ selectively excites the CO strech mode on the bridge site from the ground state to the 1st excited state, and the broadband probing beam with $\boldsymbol{\omega}_{CO_2}$ detects the population of CO on the1st excited state of the bridge site. (c) 2DIR spectrum collected at a time delay of 50 ps. (d) Representation of the peak ($\boldsymbol{\omega}_{CO_2}$,$\boldsymbol{\omega}_{CO_1}$) in supplymental figure 7c. The pumping IR light with frequency $\boldsymbol{\omega}_{CO_2}$ selectively excites the CO strech mode on the bridge site from the ground state to the 1st excited state, and vibrational energy transfers from the bridge site to the step atop site and the excited step atop site strech mode to the 1st excited state after 50ps, the broadband probing beam with $\boldsymbol{\omega}_{CO_1}$ detects 1st population of atop step site.

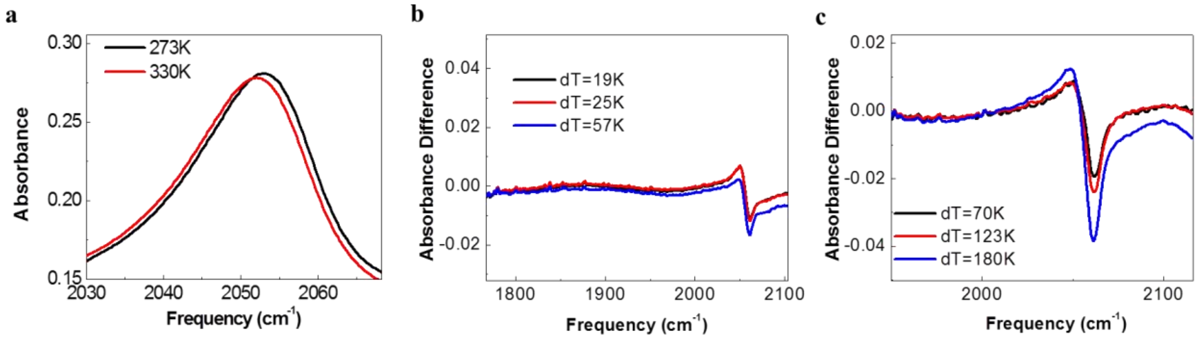

Supplementary Figure 8 Spectra of CO on step under different conditions. (a) FTIR of the CO step site collected at two temperatures. (b) Temperature difference FTIR of CO on the 5nm Pt nanoparticle. The background temperature is 273K. Here we assume that the spectral changes for the same amplitude of temperature changes are very similar for background temperatures 273K and 295K (our experimental temperature). (c) Temperature difference FTIR of CO on 5nm Pt nanoparticle. The background temperature is 150K.

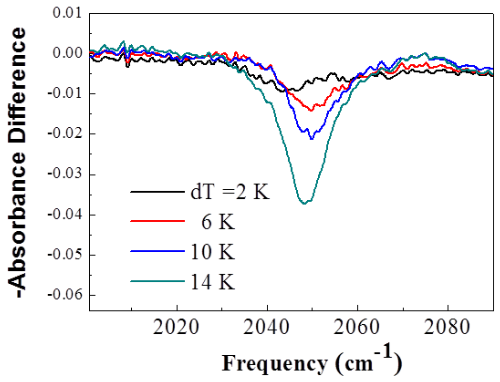

Supplementary Figure 9: Temperature dependent spectra differences of 1nm Pt CO. Temperature dependent FTIR difference spectra of CO on the 1nm Pt nanoparticle step site. The background temperature is 293K (DMF background was subtracted).

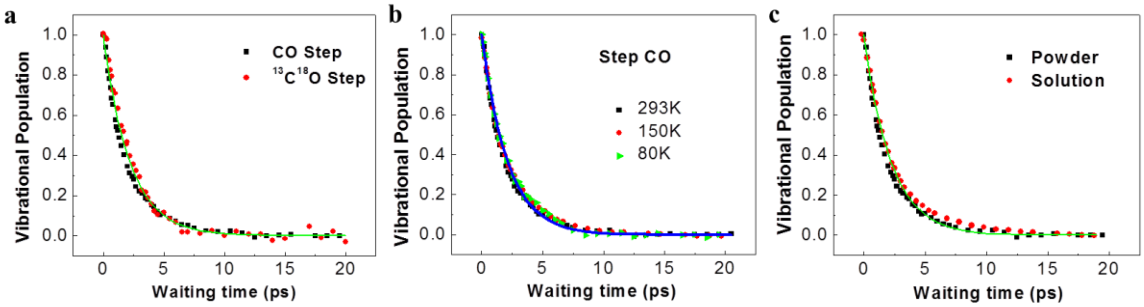

Supplementary Figure 10: CO relaxation under different conditions. (a) Vibrational relaxation decay of CO and the isotope $^{13}C^{18}O$ on the step site of the 2nm Pt nanoparticles; (b) vibrational relaxation decay of CO on the step site of the 2nm Pt nanoparticle sample at 80K, 150K and 293K; (c) vibrational relaxation decay of CO on the step site of 2nm Pt sample in powder and ethylene glycol solution.

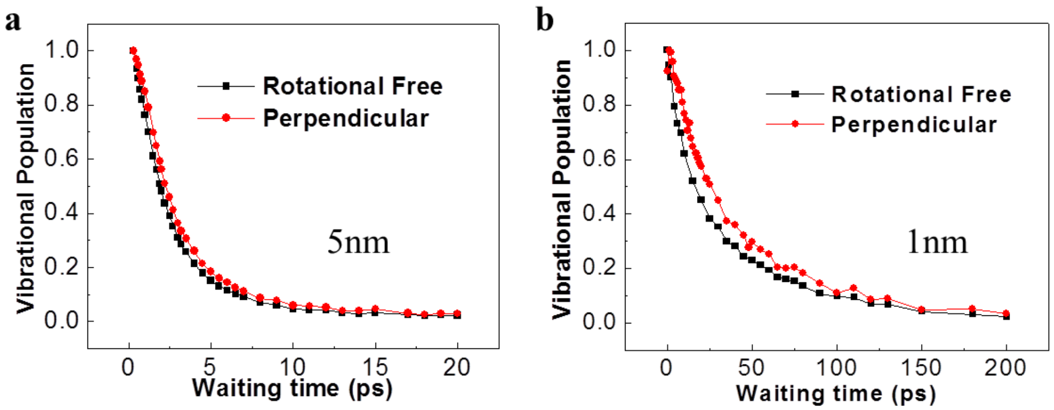

Supplementary Figure 11: CO relaxation decay under different measurements. Vibrational relaxation of CO results on (a)5nm Pt sample and the (b)1nm Pt sample are shown under perpendicular and rotational free measurements. Black is the rotational free data (parallel signal plus two times the perpendicular signal). Red is the signal from the perpendicular polarization configuration.

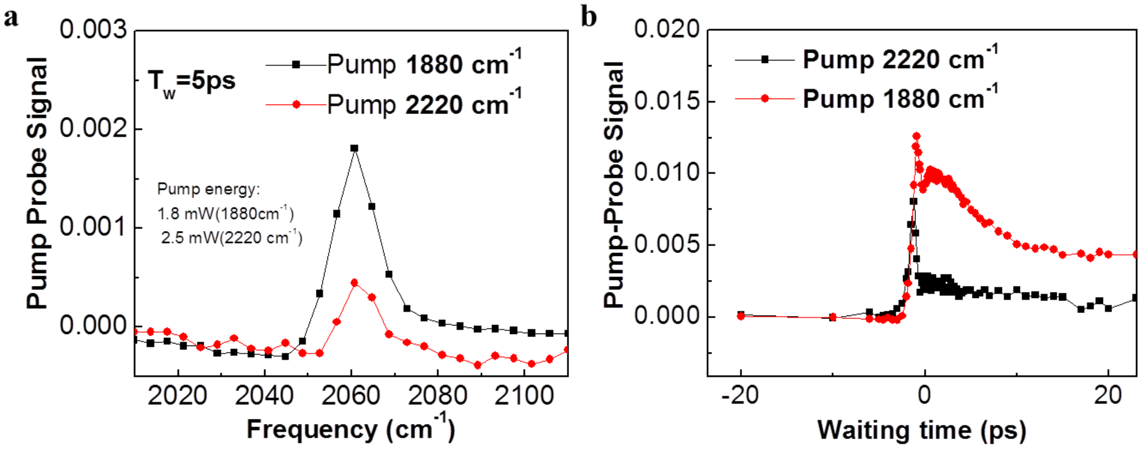

Supplementary Figure 12: Pump Probe data of 5nm Pt sample. (a) Spectra of CO stretch on the step atop site of 5nm Pt 5ps after pumping the sample with 1880 cm^{-1} (bridge CO absorption) and 2220 cm^{-1} light (control background). (b) Dynamics at 2062 cm^{-1} after pumping the sample with 1880 cm^{-1} and 2220 cm^{-1} light.

Supplementary Note 1

Direct energy exchange on 1nm Pt nanoparticle sample

In our experiments, CO molecules on different 1nm Pt nanoparticles cannot exchange energy because of low sample concentration (30μM). The excited CO on bridge [CO^*_{Bridge}] could decay with $K_{CO*Bridge}$ or transfer energy to atop CO with $K_{Bridge\rightarrow Atop}$. Meanwhile, the excited CO on atop [CO^*_{Atop}] could decay with $K_{CO*Atop}$ or transfer energy to atop CO with $K_{Atop\rightarrow Bridge}$. Thus, the time-dependent excited CO population could be presented as following:

$$\frac{d_{CO*_{Bridge}}}{dt} = -(K_{CO^*_{Bridige}} + K_{Bridge\rightarrow Atop})\left[CO^*_{Brdige}\right] + K_{Atop\rightarrow Bridge}\left[CO^*_{Atop}\right] \qquad (1)$$

$$\frac{d_{CO^*_{Atop}}}{dt} = -(K_{Atop\rightarrow Bridge} + K_{CO^*_{atop}})\left[CO^*_{atop}\right] + K_{Bridge\rightarrow Atop}\left[CO^*_{Bridge}\right] \qquad (2)$$

The kinetic calculation parameters:

$$k_{CO_{Atop}fast} = 1/10.6\ (ps^{-1});\ k_{CO_{Atopslow}} = 1/75.0\ (ps^{-1});\ k_{CO_{Bridge}slow} = 1/42.0\ (ps^{-1});$$
$$k_{CO_{Atop}\rightarrow CO_{Bridge}} = 1/180(ps^{-1});\ \text{D=0.4}$$

with pre-factors of the subgroups and offset of the bi-exponential

$$A_{CO_{atop}fast} = 0.46;\ A_{CO_{atop}slow} = 0.54; A_{CO_{bridge}fast} = 0.0;\ A_{CO_{bridge}slow} = 1.00;\ offset = 0\ .$$

For the solutions, the detailed balance is $\frac{K_{Bridge\rightarrow Atop}}{K_{Atop\rightarrow Bridge}} = 0.4$ or $\frac{K_{Atop\rightarrow Bridge}}{K_{Bridge\rightarrow Atop}} = 2.5$ due to the energy mismatch of ~187 cm^{-1}. In Fig. 2d of the main text, the bridge site 1-2 transition splits into two blue peaks (1860cm^{-1}, 1836cm^{-1}) and (1860cm^{-1}, 1810cm^{-1}). It is believed to be caused by a Fermi resonance between the bridge CO stretch 2nd excited state and some dark mode. A similar phenomenon was thoroughly described previously.[1]

Supplementary Methods

Signal Origins of our ultrafast methods

Our experimental 0-1 transition signal (diagonal red peak) comes from the ground state bleaching and the 1st excited state stimulated emission of the CO stretch mode. The 1-2 transition (diagonal blue peak) comes from the 1st excited state absorption. The 1st excited state absorption and stimulated emission signals are directly proportional to the population of the 1st excited state, and therefore their decay dynamics directly reflect the 1st excited state population decay dynamics. The ground state bleaching signal reflects the population change on the ground state: (1) the increased population on the ground state, which is equal to the decreased population on the 1st excited state; and (2) the decreased population caused by increased temperature. Because of these origins, the 1-2 transition signal in our experiments mostly reflects the vibrational relaxation dynamics, while the 0-1 transition signal is dominated by the vibrational relaxation dynamics with a small heat effect at long waiting times. This can be seen in the pump/probe data that show the red peaks last slightly longer than the blue peaks. The mechanism has been described previously in our review article.[2]

On the Pt nanoparticle surfaces, once CO molecules have been vibrationally excited, they are in the 1st excited state. In other words, the unstable CO populations in 1st state need to decay to the ground state through certain channels. There are several possible dynamic events which can affect the vibrational excitation signal decay: (1) the excited CO molecule can desorb and never come back, resulting in signal decay. This event can be precluded since we did not observe any signal loss for every repeated experiment, of which the repeating time was about one minute. (2) The excited CO molecule detach or migrate to other sites and come back after tens of nanoseconds that our experimental delay didn't reach but before 1 microsecond (ms) which is

our laser repetition rate. This scenario is not likely either because it would have produced a long-lived bleaching at the CO 0-1 transition peak that would decay much slower than the 1-2 transition. Results in Fig. 4b~d in the main text show that the CO stretch 0-1 and 1-2 transitions decay in very similar fashions. (3) The excited CO molecule can migrate back and forth on different surface sites. This is not likely either because fig.2b shows that there are no chemical exchange cross peaks.[2] (4) The vibrational excitation of the CO molecules on the step sites transfers to molecules on other sites[3]. This is not likely either because no energy transfer cross peaks appear in Fig. 2b. In conclusion, the experimental results suggest that the observed fast signal decays in Fig. 4b~d of main text occur because of the fast relaxations of CO stretch vibrational excitations.

In this paragraph, we present how the signal from the cross peak in Fig. 2a and 2b relates to heat generation on metal nanoparticle surfaces. The cross peak on the 5nm Pt sample includes contributions from both the background signal and the heat generation from the CO vibrational relaxation. The background signal comes from two sources; the first is scattered light and the second is the direct excitation of the surface electron/hole pair by the IR pulse. To obtain the pure heat generation signal from the CO vibrational relaxation, we did control experiments as shown in Supplementary Fig.12. In the control experiments, the central frequency of the IR excitation pulse was tuned to 2220 cm^{-1} where there is no vibrational resonance. The frequency mismatch between 2220 cm^{-1} and 2053 cm^{-1} (the CO stretch on the step atop site) is similar to the frequency mismatch between 2053 cm^{-1} and 1880 cm^{-1} (the CO stretch 0-1 transition frequency on the bridge site). The signal comes from the cross peak (ω_{pump}=2220 cm^{-1}, ω_{probe}=2062 cm^{-1}) including the two background signals described above. The heat generation signal from the bridge CO vibrational relaxation was obtained by subtracting the intensity of the cross peak

(ω_{pump}=1880 cm^{-1}, ω_{probe}=2062 cm^{-1}) from that of the cross peak (ω_{pump}=2220 cm^{-1}, ω_{probe}=2062 cm^{-1}) normalized with the excitation power.

Supplementary Fig. 8 displays the spectra of the step site CO at different temperatures. It is clear that the CO frequency shifts to lower frequencies at higher temperatures. The temperature increase produces a bleaching at around 2062 cm^{-1} and absorption around 2047 cm^{-1}, showed in Fig. 3b. These spectral changes also show up in the 2D IR spectra in fig.2b in the main text. Such heat-induced bleaching and absorption signals in the 2D IR measurements have been described in great detail in our previous publications.[2,4-6] The spectral changes caused by temperature increase in the frequency range of the bridge site CO stretch (~1880 cm^{-1}) are very small. The heat generation induced cross peaks around (2053 cm^{-1}, 1880 cm^{-1}) are too weak to be observed because of this as well as the overlap with the tail of the very strong 1-2 absorption signal of the step atop site CO stretch (2D IR spectra in fig.2b). The spectral resolution is about 4 cm^{-1} in our experiments.

We can additionally estimate the range of temperature increase on the particle surface. The electron/hole pair excitation caused by the vibrational relaxation occurs within 2ps and then transfers to the lattice vibration of Pt-Pt within ~1ps. If we assume that the heat (lattice vibration) transfer speed is similar to the speed of sound in platinum (1730-3260 m/s), at 25ps the energy from the CO vibrational relaxation must already distribute on the entire 5nm particle and the temperature on every location of the particle surface must be similar. Therefore, the spectral changes at 25ps in the 2D IR spectrum can be used to estimate the particle surface temperature by comparing to the temperature difference FTIR spectra. As we can see from fig.3b in main text, the spectral changes at 25ps are very similar to those in the FTIR temperature difference (25K) spectrum. This indicates that the temperature increase on the particle surface 25ps after the

CO stretch is vibrationally excited about 25 degrees. At a time delay of 2.5 ps, there are only a few local Pt atoms (probably fewer than 50 Pt atoms, within 1-2 nm radiuses) that can be heated up by the CO vibrational relaxation. The local temperature is much higher than that at 25ps. As fig.3b shows, the surface temperature increases more than 57K (At a higher temperature, the relative ratio of the absorption at ~2047 cm^{-1} to the bleaching at ~2062 cm^{-1} is smaller, as shown in Supplementary Fig. 8c).

Supplementary References

1 Zheng, J. *et al.* Accidental vibrational degeneracy in vibrational excited states observed with ultrafast Two-Dimentional IR vibrational echo spectroscopy. *J Chem Phys* **123**, 164301 (2005).
2 Chen, H., Bian, H., Li, J., Wen, X. & Zheng, J. Ultrafast multiple-mode multiple-dimensional vibrational spectroscopy. *Inter Rev Phys Chem* **31**, 469-565 (2012).
3 Backus, E. H. G., Eichler, A., Kleyn, A. W. & Bonn, M. Real-time observation of molecular motion on a surface. *Science* **310**, 1790-1793 (2005).
4 Chen, H., Bian, H., Li, J., Wen, X. & Zheng, J. Relative Intermolecular Orientation Probed via Molecular Heat Transport. *J Phys Chem A* **117**, 6052-6065 (2013).
5 Bian, H. T., Wen, X. W., Li, J. B. & Zheng, J. R. Mode-specific intermolecular vibrational energy transfer. II. Deuterated water and potassium selenocyanate mixture. *J Chem Phys* **133**, 034505 (2010).
6 Bian, H. T., Zhao, W. & Zheng, J. R. Intermolecular Vibrational Energy Exchange Directly Probed with Ultrafast Two Dimensional Infrared Spectroscopy. *J Chem Phys* **131**, 124501 (2009).
